# Predictors of Acute Myocardial Infarction: A Machine Learning Analysis After a 7-Year Follow-Up

**DOI:** 10.3390/clinpract15040072

**Published:** 2025-03-31

**Authors:** Marco Casciaro, Pierpaolo Di Micco, Alessandro Tonacci, Marco Vatrano, Vincenzo Russo, Carmine Siniscalchi, Sebastiano Gangemi, Egidio Imbalzano

**Affiliations:** 1 Allergy and Clinical Immunology Unit, Department of Clinical and Experimental Medicine, University of Messina, 98125 Messina, Italy; mcasciaro@unime.it (M.C.); gangemis@unime.it (S.G.); 2 UOC Medicina Interna, AFO Medica, P.O. Santa Maria delle Grazie, ASL Napoli 2 Nord, 80076 Pozzuoli, Italy; pdimicco@libero.it; 3 Institute of Clinical Physiology, National Research Council of Italy (IFC-CNR), Via G. Moruzzi 1, 56124 Pisa, Italy; alessandro.tonacci@cnr.it; 4 Cardiology Unit, “Pugliese-Ciaccio” Hospital, 88100 Catanzaro, Italy; marco.vatrano1975@gmail.com; 5 Cardiology Unit, Department of Translational Medical Sciences, University of Campania “Luigi Vanvitelli”-Monaldi Hospital, Piazzale Ettore Ruggeri, 80131 Naples, Italy; vincenzo.russo@unicampania.it; 6 Department of Continuity of Care and Multicomplexity, Azienda Ospedaliero-Universitaria di Parma, 43126 Parma, Italy; 7 Department of Clinical and Experimental Medicine, University of Messina, 98125 Messina, Italy; egidio.imbalzano@unime.it

**Keywords:** myocardial infarction, heart, machine learning, predictors, artificial intelligence

## Abstract

**Background:** Ischemic heart disease is a major global health problem with significant morbidity and mortality. Several cardiometabolic variables play a key role in the incidence of adverse cardiovascular outcomes. **Objectives:** The aim of the present study was to apply a machine learning approach to investigate factors that can predict acute coronary syndrome in patients with a previous episode. **Methods:** We recruited 652 patients, admitted to the hospital for acute coronary syndrome, eligible if undergoing immediate coronary revascularization procedures for ST-segment-elevation myocardial infarction or coronary revascularization procedures within 24 h. **Results:** Baseline pulse wave velocity appears to be the most predictive variable overall, followed by the occurrence of left ventricular hypertrophy and left ventricular end-diastolic diameters. We found that the potential of machine learning to predict life-threatening events is significant. **Conclusions:** Machine learning algorithms can be used to create models to identify patients at risk for acute myocardial infarction. However, great care must be taken with data quality and ethical use of these algorithms.

## 1. Introduction

Ischemic heart disease (IHD) is a major global health problem with significant morbidity and mortality, accounting for 20% of all deaths in Europe [1,2]. The etiology of IHD is identified as a consequence of the interaction between several risk factors, including lifestyle and genetic risk factors [3]. One of the most common causes of acute myocardial infarction (AMI) is obesity, which is widely recognized as one of the most important modifiable causes of cardiovascular (CV) morbidity and mortality [4], mainly due to its adverse effects on metabolic components [5]. It is most commonly defined through the “body mass index” (BMI) and plays a key role in metabolic syndrome (MetS) according to the American Heart Association/National Heart, Lung and Blood Institute criteria [6]. Recent reports have identified some subpopulations with different BMI and metabolic characteristics, differing in weight and healthy or unhealthy metabolic profile [7,8], with different risks of type 2 diabetes mellitus and cardiovascular disease [9]. Other reports also confirm a close correlation between older age and higher risk of CVD in this type of population [10]. Although IHD is one of the most studied cardiovascular diseases, its complex mechanism is still a matter of debate due to its different characteristics in different populations. Acute coronary syndrome (ACS) seems to occur four times more often in younger men than in younger women (under 60 years old), and vice versa; after 75 years old, women represent the majority of patients [11]. The current literature shows a non-direct relationship between metabolic profile and cardiovascular events; on the contrary, it seems that various cardiometabolic variables play a key role in the incidence of adverse cardiovascular outcomes [1,12,13,14,15]. Conflicting results are described in the metabolically unhealthy obese (MUO) population, which seems to have a higher incidence of CV diseases with a higher risk of mortality and morbidity compared to the metabolically healthy obese (MHO) population, and the latter confers an increased risk of heart failure, but not of acute myocardial infarction. To our knowledge, the effects of the metabolic phenotype on CV recurrence are unknown in patients with a previous AMI treated with coronary revascularization procedures. Previous research indicates several predictors of AMI, from demographic to socioeconomic, geographic, gender-related, and psychological factors [16,17]. The aim of this research was to investigate the risk factors for acute coronary syndrome, in particular those related to body mass index, in patients with previous ACS by using a machine learning approach.

## 2. Materials and Methods

### 2.1. Population

We recruited 652 patients (Table 1), admitted to the University Hospital “G. Martino” of Messina, hospitalized for acute coronary syndrome (ACS). Patients were eligible if they met the criteria for ACS, proposed by the Joint European Society of Cardiology/American College of Cardiology Committee [1,5], and if they underwent immediate coronary revascularization procedures (primary PCI) for ST-elevation myocardial infarction (STEMI), coronary revascularization procedures (early PCI) within 24 h for myocardial infarction without ST elevation (nSTEMI), or diagnostic coronary angiography following coronary artery bypass graft surgery (CABG).

Exclusion criteria were well-known coronary artery disease or previous ACS, PCI following CABG, cardiogenic shock, atrial fibrillation, peripheral artery disease, severe cardiac valve disease, and prosthetic aorta.

### 2.2. Variables Measurements

Variables investigated were blood pressure, metabolic profile, and body mass index. Also, transthoracic echocardiography was performed for each patient. Blood pressure (BP) was obtained in the supine position after five minutes of quiet rest with an aneroid sphygmomanometer, and definite values were obtained as the means of three consecutive measurements obtained every 3 min. Patients with a clinical SBP > 140 mm Hg and/or DBP > 90 mm Hg were defined as hypertensive. Body mass index (BMI) was obtained as body weight (kilograms) divided by the squared height (meters) and further subdivided into the normal weight category if BMI was <25 kg/m^2^, overweight if BMI was 25.1–29.9 kg/m^2^, and obese if BMI was >30 kg/m^2^. Metabolic status was measured as the circumferences of the waist and hips while the patient was standing and with his arms relaxed. We also examined fasting serum samples for glucose and lipid profiles. Then, according to the definition of metabolic syndrome (MetS) of International Diabetes Federation, we defined the disease if three of five components were registered: (1) blood pressure (BP) ≧ 130/85 mmHg or drug treatment for elevated blood pressure; (2) a waist circumference (WC) ≧ 90 cm (in men) and ≧80 cm (in women) or BMI ≧ 30 kg/m^2^; (3) fasting plasma glucose (FPG) ≧ 100 mg/dL or drug treatment for elevated blood sugar; (4) serum triglycerides (TG) ≧ 150 mg/dL or drug treatment for elevated triglycerides; (5) serum high-density lipoprotein (HDL) < 40 mg/dL (in men) and <50 mg/dL (in women) or drug treatment. Transthoracic echocardiography (TTE) was performed by expert sonographers who evaluated the patients without knowing their blood pressure or other clinical data. This involved VIVID 7 ultrasound machines (GE Technologies, Milwaukee, WI, USA) with an annular phased array 2.5 MHz transducer, as recommended by the American Society of Echocardiography.

### 2.3. Population Categories

In order to categorize all participants according to the presence or absence of MetS or obesity, we divided them into four groups: metabolically healthy and normal weight (MHNW), metabolically unhealthy but normal weight (MUNW), metabolically healthy but obese (MHO), and metabolically unhealthy and obese (MUO). Written informed consent was obtained from each participant before initiating any study-related procedure.

### 2.4. Study Outcomes

Patients were followed from coronary revascularization for 7 years. Clinical out-comes were evaluated by monitoring major adverse cardiovascular events (death, fatal, or non-fatal re-infarction with or without PCI and stroke), seen as cumulative cardiovascular (cCV) events for all statistical analyses. Procedure-related AMI within 24 h was excluded in the endpoint.

### 2.5. Machine Learning: General Principles

Machine learning (ML) was employed to predict the clinical outcomes of patients included in the dataset based on the input data collected. The clinical outcome prediction was represented by the occurrence of a hospital admission within a 7-year follow-up. The presence/absence of the above-mentioned event requires a classification task, for which suitable models should be implemented and applied. For such models, the best performances were selected according to the accuracy of the prediction (i.e., the number of correct predictions with respect to the “real” values of the outcome). However, other parameters, including the time needed to train the models and the best fit hyperparameters, will be mentioned in the results.

For all the models, the dataset was divided into 70% of data employed for model training (i.e., the training set) and 30% for model testing (i.e., the test set). All the models were evaluated on 10-fold cross-validation, in order to reduce overfitting, ensuring that the model possessed enough generalizability to further unknown data.

In terms of the variables used for the analysis, a general linear model (GLM) was first set up. The variables with significance in this first analysis, featuring a *p*-value below *p* = 0.10, were checked for multicollinearity and further selected for application in the models.

The whole ML analysis was carried out under the open-source R language using the software RStudio, version 2022.12.0 Build 353 for Windows, available with the GNU Affero General Public License. All the models were run on a laptop equipped with AMD Ryzen 5 3500U with Radeon Vega Mobile Gfx at 2.10 GHz.

### 2.6. Machine Learning Models

For the present work, three models suitable for classification purposes were employed to predict each of the outcomes. Given the prior knowledge of the output values, the models selected were “supervised models” and included classification and regression trees (CARTs), random forest (RF), and support vector machines (SVMs). The models are briefly presented below.

#### 2.6.1. CART

Classification and regression trees (CARTs) are popular, powerful ML models. Their bedrock relies on the deconstruction of the overall dataset into smaller groups, via binary splits of the sample, repeated, one exploratory variable at a time. CARTs outperform other models in their speed of execution and their adaptation to nearly all kinds of data (cross-sectional, longitudinal, and survival data), without requiring to be distributed in a Gaussian fashion. At the same time, they can be used both for classification (as in this case) and regression purposes. On the other hand, they are quite sensitive to small data changes and present limited interpretability.

#### 2.6.2. Random Forest

Random forests (RFs) are very popular ML models that can be applied to both regression and classification tasks. They also rely on decision trees for training, but a much higher amount, making up a “forest” of trees. Like the “classical” decision trees, they do not need any particular data preparation prior to the application of the model; they can process binary, categorical, and numerical features without any need for scaling, normalization, or standardization. The advantages they have with respect to the “classical” decision trees include the performance of implicit on-the-run feature selection and the provision of more accurate indicators of feature importance. Finally, they are unlikely to perform overfitting and they are relatively quick to train and versatile, but their interpretability is questionable.

#### 2.6.3. SVM

Support vector machines (SVMs) are robust, popular methods for prediction, both adapted to classification and regression purposes. When receiving a set of training observations, with a labeled category, the SVM algorithm builds a model assigning new observations to one category or another, mapping the training observations to points in the space, aimed at maximizing the gap between the categories. New observations are then mapped and predicted based on the side of the gap they fall on. To perform this, different kinds of classifications are possible, including linear (the most popular and simplest one) or non-linear ones, mapping inputs into high-dimensional feature spaces, with the application of dedicated kernels (radial, polynomial, sigmoid, etc.). For the present study, in order to manage the trade-off between prediction accuracy, computational load, and execution speed, we evaluated the performances of linear and radial basis function (RBF) kernels using a grid search for hyperparameter tuning.

## 3. Results

During the 7-year follow-up period, patients underwent two follow-up visits in the first year and annual visits thereafter. The incidence rate of the assessed endpoint varied among the different metabolic and obesity status groups: 8.4 per 100 patient-years in the MHNW (metabolically healthy normal weight) group, 14.6 per 100 patient-years in the MUNW (metabolically unhealthy normal weight) group, 12.5 per 100 patient-years in the MHO (metabolically healthy obese) group, and 20.4 per 100 patient-years in the MUO (metabolically unhealthy obese) group. These findings highlight a higher incidence of the endpoint in metabolically unhealthy and obese patients, suggesting a potential impact of metabolic status and obesity on long-term outcomes after revascularization. We find that variables associated with hospital admission included metabolic obesity, obesity, metabolic healthy normal weight (MHNW), height, weight, pulse wave velocity (PWV), first stent length, intervascular (IV) septum size, posterior wall size, E/A ratio (the ratio between early and late transmitral flow), and the presence of metabolic syndrome at baseline. In terms of the models employed, the results related to the test set accuracy are displayed in Table 2.

Random forest was seen to perform the best, correctly classifying data in 85.15% of cases in a relatively short amount of time (less than 3 min with the above-mentioned computational availability). However, if we consider sensitivity and specificity as other parameters of interest, then random forest holds an excellent 89.25% of specificity; however, it falls to 37.50% in terms of sensitivity, failing in classifying as positive most of the real positive cases despite the excellent performances reported with the training set, where all individuals were correctly classified. This lack of performance in the test set was seen in all the models and is likely due to different factors, among which is the relatively scarce number of real positive cases in the test set, which is deemed an intrinsic issue of the present dataset.

**Figure 1:**
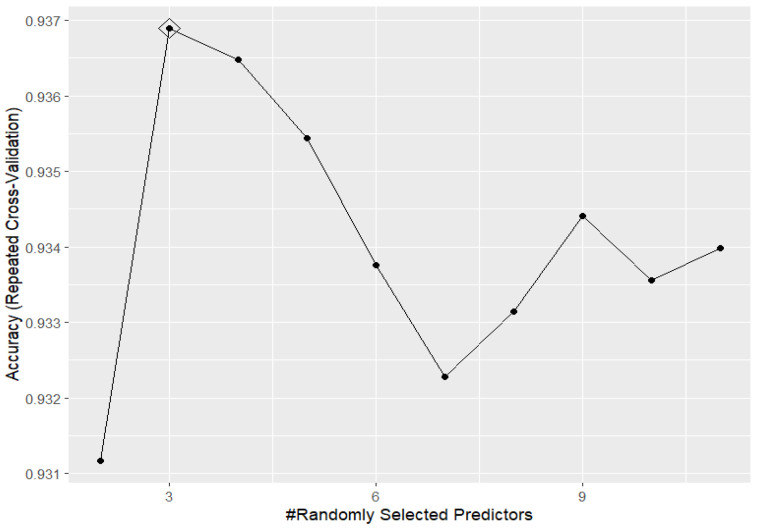
Accuracy performances, varying the mtry hyperparameter, for random forest in predicting the outcome based on the training set data.

Focusing on the “best model” in this task, random forest was seen to perform the best when the mtry parameter, i.e., the number of predictors randomly sampled at each split when creating the tree models, was set to be equal to 3 (see Figure 1).

This means that the model is quite simple overall and explores just a very small subset of the features contained in the dataset, with most of the features included in it deemed not predictive for the specific classification task required.

However, artificial intelligence is now becoming easier and easier to interpret, even by people without particular skills in information technology (IT) and close subjects. The research on the so-called explainable artificial intelligence (explainable AI, or XAI) is rapidly growing, and even the medical sector is interested in this scaling up of data interpretation. Of course, XAI also makes use of approaches and tools, which are out of the scope of the present article; however, the clinician might strongly benefit from a brief interpretation of the obtained results.

**Figure 2:**
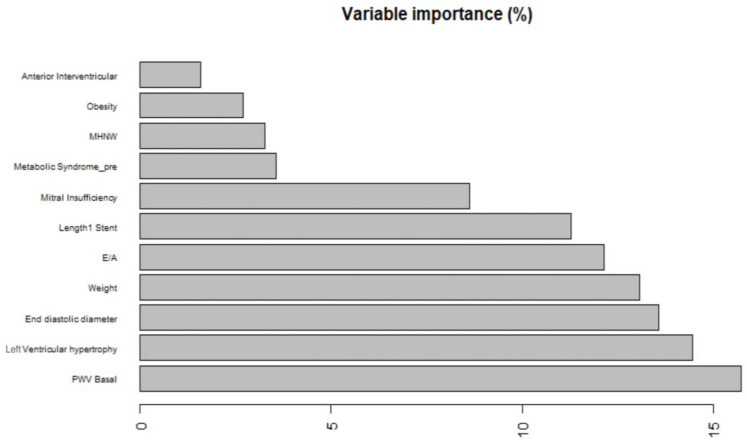
Variable importance, as a percentage of the overall relative weight, for the random forest model. MHNW: metabolically healthy normal weight; PWV Basal: pulse wave velocity at baseline; E/A: ratio of early (E) to late (A) ventricular filling velocities (a marker of diastolic function).

One of the first evaluations to be performed in this sense is related to the study around the variable importance in outcome prediction. The analysis performed gave the results shown in Figure 2.

As displayed in the figure, the basal value of the pulse wave velocity (PWV) appears to be largely the most predictive variable overall, followed by the occurrence of left ventricular hypertrophy and left ventricular end-diastolic diameters. The basal value of pulse wave velocity is recorded as the velocity at which the mechanical wave propagates along the arterial wall. PWV represents a useful surrogate marker for arterial vessel stiffness. With respect to the other models trained and whose parameters are not shown here due to their worse performances compared to random forest, the latter does give a lower importance to the E/A ratio, i.e., the ratio between early and late transmitral flow. This parameter does not appear to be as predictive as supposed by the CART, giving an important insight for the clinician in the interpretation of the available data, possibly also influencing the choice of certain exams in the basal clinical characterization of an individual with early suspected evidence for a possible future cardiovascular event that might lead to hospital admission.

Another important consideration that could be derived regards the “computational load” for such models. In all cases, they were run on a modern, although average-priced laptop, an instrument that is affordable for most end-users. This choice for a bench testing strategy was performed to evaluate the feasibility of running such models on general-purpose machines, so that the results obtained can be scalable to nearly any domain, including everyday clinical practice. Referring to this, random forest, despite being the slowest model to provide results given its relative complexity with respect to the CART and SVM, completed the task within 3 min from its launch, proving the ability to perform a fair estimation of the outcome even in very short amounts of time, highlighting its usability even in non-laboratory settings or in disadvantaged places around the globe, where computational resources are often a major limitation to AI exploitation. In cases where superior performances in terms of computational speed are required, several research infrastructures devoted to calculation can be reached through dedicated agreements, such as, for example, the SoBigData++ (https://plusplus.sobigdata.eu/) infrastructure, hosted at the National Research Council of Italy, granting faster response times and optimal computational performances overall.

## 4. Discussion

In recent years, many studies have shown machine learning (ML) applications in medical research [18,19]. In fact, ML can be used as a standalone application, but even more proficiently, alongside traditional risk scores to predict the occurrence of acute events [20]; to follow disease trajectories [21], allowing for precise risk stratification; or in decision support systems (DSSs) [22], to the benefit of the clinician. With respect to the current literature, although our best performing model is deeply affected by quite a low sensitivity, which is a major drawback for prediction purposes in the clinical setting—especially with AMI, being that such a result was probably affected by the much lower occurrence of “positive” cases in our dataset—our findings are confirmed by many reports in the literature about the association between pulse wave velocity (PWV), left ventricular hypertrophy (LVH), left ventricular end-diastolic diameter (LVEDD), and the increased risk of AMI, confirming their role in the incidence of acute coronary syndrome [23,24]. Studies have shown that PWV, a non-invasive measure of arterial stiffness, is strongly correlated with cardiovascular disease and mortality. It is believed to be a marker of the aging process, increasing in older people, and is associated with the development of atherosclerosis and hypertension [25]. Many reports also showed PWV as an independent predictor of cardiovascular events. In particular, it was found to be a significant predictor of AMI, stroke, and heart failure [26], even more than the known traditional risk factors such as blood pressure and cholesterol levels [27]. Many studies also hypothesized to define PWV cut-off values to improve individual risk stratification [28,29]. A study on 136 consecutive patients who were diagnosed with ST-segment-elevation coronary syndrome and treated with primary percutaneous coronary intervention showed that PWV values recorded within 48 h after acute MI were able to predict LVEF changes at 6 months from ACS, thereby obtaining important information during the follow-up [30,31]. According to the literature, left ventricular hypertrophy, as we can show in our study, seems to be correlated with a higher risk of AMI [32,33]. Similar to the organ damage caused by many cardiovascular diseases such as hypertension and diabetes, it also underlines microvascular injury, leading in turn to major cardiovascular events such as acute myocardial infarction, making it a strong predictor of CVD [34,35]. Furthermore, in a sub-study of the DANAMI 3-trial on 764 STEMI patients treated with primary percutaneous coronary intervention, it was demonstrated that LVH is independently associated with a larger infarct size, less myocardial salvage, a higher incidence of microvascular obstruction, a lower LVEF, and a higher risk of all-cause mortality and the incidence of heart failure [36]. Although it is known that LVH is mostly associated with CAD in hypertensive patients, the literature also reports an association between CAD and normotensive patients, considering LVH as a pro-inflammatory factor that would promote atherosclerosis in all vascular systems, such as coronary and carotid arteries. Therefore, LVH can be a marker in patients with ischemic heart disease, developing arrhythmia due to atherosclerosis, endothelial dysfunction, or remodeling [37,38]. End-diastolic diameter, as a predictor of cardiovascular events [39], was demonstrated to be predictive for AMI and hospitalization from the machine learning analysis. In a study of 1459 patients admitted for acute coronary syndrome, diastolic function, left ventricular end-diastolic pressure, and diameter seems to have a significant prognostic influence in patients with ACS regarding future readmission due to congestive heart failure. Nevertheless, the role of LVEDD calculation in outcome prediction is not debatable; however, further studies are needed to investigate the thorough task of treatment strategies in post-infarcted patients. Furthermore, another study confirms that left ventricular remodeling (LVR) development was associated with a higher risk for adverse composite outcomes and HF rehospitalization, especially in patients with STEMI with multivessel disease [40].

**Figure 3:**
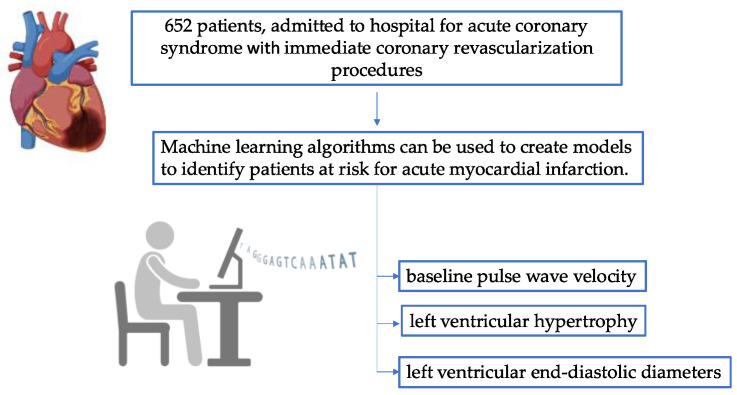
Model and results.

So, we can confirm that the correlations among PWV, LVH, and LVEDD with the risk of hospital admission for AMI are complex. All three parameters are markers of underlying vascular damage that leads to health impairment. AMI is often an outcome of this harm. However, the exact correlation among these measures is not straightforward, and their relationship will be for the subject of further investigations. Other authors found a link between PWV and LVH. In a study on patients affected by hypertension, PWV was found to be a predictor of VH [25]. Another study found that there was a correlation between PWV and left ventricular mass [28]. Furthermore, a correlation between VH and LVEDD appears to be present, too. In fact, in a cohort of individuals with hypertension, LVH was found to be a predictor of left ventricular diastolic dysfunction, as measured by LVEDD [41]. PWV, LVH, and LVEDD will help in identifying subjects who have a high risk for the recurrence of cardiovascular events. Monitoring these patients who would benefit from early intervention, such as lifestyle modifications, medication, or surgery, is the aim of the physicians. However, further research is needed to confirm the predictive efficacy of the combination of these measures on AMI recurrence. Figure 3 summarizes the model in a simple way.

### Limitations and Future Directions

The results displayed in the present paper should be considered in light of some study limitations. Despite the relatively large dataset considering the topic evaluated, the balance between positive cases (i.e., the patients effectively requiring hospital admission within the timeframe selected) and negative ones (i.e., those not requiring such intervention) was far from optimal, as the patients admitted were just below 10% of the overall cases, representing a significant problem for ML algorithms trying to perform a subject classification. In the future, this might be overtaken by including a larger number of patients experiencing a related hospital admission and applying approaches to limiting the class imbalance, like the synthetic minority over-sampling technique (SMOTE) or random over-sampling (ROS). Second, as happens in a good number of studies, the dataset is composed of patients from a single clinical center. This might affect the overall generalizability of the results obtained, which might depend on the methodologies with which some of the parameters are acquired. Future studies should therefore improve this aspect by enrolling patients from different clinical centers and allowing an external validation of the results obtained using different datasets. Finally, a more complete characterization of all the patients is required. Indeed, as usually occurs in real clinical studies, the present dataset also includes a number of missing values (overall proportion around 4%), which were replaced using validated, well-established imputation methods like multiple imputation by chained equations (MICE), often applied in ML. However, more complete datasets could even improve the reliability of the data collected and the results obtained.

## 5. Conclusions

In conclusion, the correlation between pulse wave velocity, ventricular hypertrophy, and left ventricular end-diastolic diameter has been studied in relation to the risk of hospital admission for acute myocardial infarction.

These factors are significant predictors of cardiovascular events and all-cause mortality. The use of non-invasive imaging techniques such as echocardiography has enabled the accurate measurement of these parameters, making them valuable tools in the early diagnosis and prevention of acute myocardial infarction. With the advent of big data analytics and the availability of electronic medical records, machine learning algorithms can be used to identify patterns that may indicate an increased risk of acute myocardial infarction. This may allow for early intervention and prevention of cardiovascular events, ultimately leading to better patient outcomes. However, it is important to note that machine learning algorithms are only as good as the data they are trained on. The quality and accuracy of the data used to train these algorithms are critical to determining their effectiveness. It is also essential that the use of machine learning be conducted ethically and with due regard for patient privacy and autonomy. The potential of machine learning to predict life-threatening events is significant, but careful consideration must be given to the quality of the data and the ethical use of these algorithms.

## Figures and Tables

**Table 1:** Demographic, clinical, and biochemical characteristics of the study population stratified by metabolic and obesity status. We also described cardiovascular risk factors, the extent of coronary artery disease, coronary revascularization procedures, and medication use following revascularization.

	TOTAL (*652 pts*)	MHNW (*358 pts*)	MUNW (*168 pts*)	MHO (*40 pts*)	MUO (*86 pts*)	***p*-Value**
**Demographic characteristics**						
Male sex (%)	74.8	77.4	71.3	70.0	72.7	0.365
Age (year)	64.0	64.1	64.3	63.2	63.5	0.924
**Cardiovascular risk factors**						
Smokers (%)	38.6	40.2	42.1	35.0	26.1	0.062
Hypertension (%)	65.7	58.4	71.3	62.5	86.4	<0.001
Diabetes mellitus (%)	28.0	19.0	44.9	12.5	38.6	<0.001
**Clinical parameters**						
Systolic BP (mmHg)	142.6	139.0	149.7	133.6	146.3	<0.001
Diastolic BP (mmHg)	79.6	78.2	79.6	81.2	84.8	0.001
Heart rate (beats/min)	71.9	71.3	72.4	69.8	74.3	0.320
Ejection fraction (%)	53.8	54.3	52.3	56.5	54.3	0.316
E/A	0.3	0.2	0.4	0.1	0.4	0.09
**Extent of coronary artery disease at baseline**						
No significant coronary artery disease (%)	9.8	12.8	2.8	20.8	6.8	0.005
1-vessel disease (%)	36.9	37.0	39.9	35.0	31.8	
2-vessel disease (%)	24.5	24.2	27.0	20.0	22.7	
3-vessel disease (%)	28.8	26.1	30.3	25.0	38.6	
**Coronary revascularization at baseline**						
Single drug-eluting stent (%)	43.6	41.0	48.9	45.0	43.2	0.386
Multiple drug-eluting stents with overlapping (%)	12.0	12.2	12.4	7.5	12.5	0.843
Coronary artery bypass graft surgery (%)	10.5	9.8	11.2	10.0	12.5	0.568
**Biochemical markers**						
Total cholesterol (mg/dL)	181.7	179.5	185.7	184.6	181.3	0.461
HDL cholesterol (mg/dL)	44.9	49.0	36.4	52.0	42.1	<0.001
LDL cholesterol (mg/dL)	107.0	108.1	107.3	107.4	101.3	0.565
Triglycerides (mg/dL)	146.5	111.7	209.3	107.0	181.9	<0.001
Fasting glucose (mg/dL)	126.4	114.7	151.8	104.4	134.2	<0.001
Creatinine (mg/dL)	1.0	1.0	1.0	0.9	0.9	0.746
C reactive protein (mg/dL)	10.5	8.5	11.4	12.4	15.4	0.280
**Medication following coronary revascularization**						
Antiplatelet therapy (%)	99.5	99.4	99.6	99.2	100.0	0.954
Statins (%)	99.3	99.7	99.4	98.9	99.5	0.944
Diuretics (%)	28.3	27.4	27.7	28.6	29.7	0.554
ACE inhibitors (%)	97.4	97.6	96.6	98.1	97.3	0.898
Beta-blockers (%)	95.5	95.2	95.1	96.5	95.3	0.789

**Table 2:** Performances of the different models trained on the test set data.

Metrics	CART	RANDOM FOREST	SVM	
			**LINEAR**	**RBF**
Accuracy (%)	82.18	85.15	73.76	82.18
Sensitivity (%)	14.29	37.50	11.54	11.54
Specificity (%)	87.23	89.25	82.95	92.61
Time elapsed (s)	37.84	173.17	2.55	19.22
Hyperparameter (s)	cp = 0.041	mtry = 3	cost = 5 SVs = 82	Cost = 1 Gamma = 1 SVs = 124

Performances of the different models trained on the test set data (CART: classification and regression tree; cp: cost complexity parameter; mtry: number of predictors that will be randomly sampled at each split when creating the tree models; RBF: radial basis function; SV: support vector; SVM: support vector machine).

## Data Availability

Data supporting the reported results can be requested by email to the corresponding author.
